# Association between Body Mass Index and Reproductive Outcome in Women with Polycystic Ovary Syndrome Receiving IVF/ICSI-ET

**DOI:** 10.1155/2020/6434080

**Published:** 2020-08-21

**Authors:** Han Zhou, Dan Zhang, Zhuoye Luo, Aimin Yang, Na Cui, Guimin Hao, Wei Wang

**Affiliations:** ^1^Second Hospital of Hebei Medical University, Shijiazhuang, 050000 Hebei, China; ^2^The Fourth Hospital of Shijiazhuang, Shijiazhuang, 050000 Hebei, China; ^3^Cangzhou Central Hospital, Cangzhou, 061000 Hebei, China

## Abstract

**Objective:**

To examine the association between body mass index (BMI) and the outcome of in vitro fertilization or intracytoplasmic sperm injection embryo transfer- (IVF/ICSI-ET) assisted reproduction in women with polycystic ovary syndrome (PCOS) receiving the ultra-long agonist protocol.

**Methods:**

We retrospectively identified all women receiving IVF/ICSI-ET for the first time using the ultra-long protocol between January 2013 and January 2018 at our hospital. Only women at ≤35 years of age receiving the first cycle were analyzed.

**Results:**

A total of 1782 women were included in the analysis: 42 were underweight, 742 were overweight, 198 were obese, and 800 were normal weight. Gonadotropin dosage and duration were comparable between underweight and normal weight groups but were significantly higher/longer in overweight and obese groups (*P* < 0.008). The number of oocytes retrieved was significantly lower in overweight and obese groups than in the normal weight group (*P* < 0.008). The number of transferable embryos was significantly higher in normal weight group than overweight and obese groups (*P* < 0.008). Embryo implantation rate, clinical pregnancy rate, full-term birth rate, and live birth rate did not differ among the 4 groups. The cycle cancellation rate was lower in the overweight and obese group than normal weight group (*P* < 0.008). The miscarriage rate was higher in the obese group than the normal weight group (*P* < 0.008). In multivariate logistic regression analysis, abnormal BMI was an independent risk for miscarriage (OR: 1.069, 95% CI 1.020, 1.122; *P* = 0.006).

**Conclusion:**

Overweight and obesity are associated with poor outcomes in PCOS patients receiving ultra-long protocol. Measures to reduce body weight should be encouraged in overweight and obese PCOS women at ≤35 years of age prior to assisted reproductive technology (ART).

## 1. Introduction

Polycystic ovary syndrome (PCOS) is a common endocrine disorder in women of reproductive age [1, 2]. Approximately 80%-95% of women with anovulatory infertility have PCOS [3, 4]. Overweight and obesity are increasingly common in the general population as well as in women of reproductive age. Estimated age-standardized prevalence in women is 14.9% (13.6%-16.1%) for obesity (BMI ≥ 30 kg/m^2^), 5.0% (4.4%-5.6%) for severe obesity (BMI ≥ 35 kg/m^2^), and 1.6% (1.3%-1.9%) for morbid obesity (BMI ≥ 40 kg/m^2^) [5]. Obesity has been associated with adverse obstetric outcome [6]. PCOS is associated with obesity [7], 40%-70% of women with PCOS are either overweight or obese [8], and the incidence of PCOS in overweight or obese women is four times that of women with normal weight [9]. In China, approximately one-third of PCOS women have a BMI higher than 23 kg/m^2^ [10]. Additionally, obesity has been associated with adverse reproductive outcomes in Chinese women with PCOS [11].

An increasing number of PCOS women are receiving ART. However, studies on the impact of overweight and obesity on the reproductive outcome of PCOS women who undergo in vitro fertilization/intracytoplasmic sperm injection-embryo transfer (IVF/ICSI-ET) have yielded conflicting results [12]. The discrepancy among these studies could be attributed to a variety of factors, including limited sample size, racial differences, and variations in ovarian stimulation protocol [13, 14].

In this retrospective analysis, we examined the potential association between BMI with reproductive outcomes in PCOS women receiving ultra-long protocol for IVF/ICSI-ET.

## 2. Materials and Methods

### 2.1. Patients

We screened all PCOS women who received IVF/ICSI-ET for the first time between January 5, 2013, and January 29, 2018, at the Department of Reproductive Medicine, Second Clinical Hospital, Hebei Medical University (Shijiazhuang, Hebei, China). PCOS was diagnosed according to the 2003 Rotterdam consensus criteria [15]. Women aged ≤35 years who received the ultra-long follicular phase agonist protocol were included. Only the first cycles using fresh embryos were included in the analysis. We excluded (1) women with uterine malformation, endometriosis, or hydrosalpinx; (2) women who had a history of uterine and ovary surgery; and (3) women with any thyroid disease, diabetes, or autoimmune disease.

The study protocol was approved by the Ethics Committee (No. 2013L-25). Patient consent was not required due to the retrospective nature of the study. Patient data were anonymized.

### 2.2. Data Retrieval

We retrieved the following information from electronic medical records: age, duration of infertility, BMI, basal follicle-stimulating hormone (FSH), serum basal luteinizing hormone (LH), and serum basal testosterone, the type of controlled ovary stimulating protocol, and the level of estradiol (E_2_), progesterone (P), and LH, and endometrial thickness on the day of human chorionic gonadotropin (HCG) administration, the number of oocytes retrieved, mode of fertilization, the number of transferrable embryos, embryo implantation rate, as well as clinical outcomes (ectopic birth, clinical pregnancy, and live birth). Women were considered underweight if BMI was <18.5 kg/m^2^, normal weight if 18.5 kg/m^2^ ≤ BMI < 25 kg/m^2^, overweight if 25 kg/m^2^ ≤ BMI < 30 kg/m^2^, and obese if BMI was ≥30 kg/m^2^ according to the most recent BMI classification by the World Health Organization (WHO) [16].

Clinical pregnancy was defined by the observation of gestational sacs, including ectopic gestational sacs, by transvaginal echographic screening at day 30 post embryo transfer. Biochemical pregnancy was defined as a positive urine or serum HCG test, defined by >5 IU/L of serum beta-HCG at day 14 post embryo transfer, in women who received luteal support until day 14 post oocyte retrieval.

### 2.3. Controlled Ovarian Stimulation Protocols

Subjects received 3.75 mg long-acting gonadotropin-releasing hormone agonist (GnRH-a) on day 2 of the menstrual cycle to achieve the goal of FSH < 5 mIU/mL, LH < 5 mIU/mL, E_2_ ≤ 50 pg/mL, and endometrial thickness < 5 mm. Serum hormone levels, including LH, E_2_, and P, were measured and women received a B ultrasound examination 30 days after GnRH-a treatment. Follicle growth was monitored 4-5 days after rFSH (150 IU to 300 IU/day) was given. The dose and duration of gonadotropin were adjusted based on serum LH and E_2_ levels until the day of HCG administration. When the number of dominant follicles (diameter ≥ 17 mm) was ≥3, and the diameter of less dominant follicles was ≥14 mm, 6000~10000 IU HCG was given to trigger ovulation. Thirty-seven hours after HCG triggering, oocyte retrieval was performed via transvaginal ultrasound-guided follicular aspiration. Embryos were cultured using a standard protocol. Two cleavage-embryos were transferred three days after oocyte retrieval under ultrasound guidance. Luteal support (progesterone injection 40 mg plus dydrogesterone tablet 20 mg/day, or sustained-release progesterone vaginal gel 90 mg plus dydrogesterone tablet 20 mg/day) was provided after oocyte retrieval and continued if pregnancy ensued and then tapered until discontinuation. Pregnant women received regular prenatal checkups until deliveries. Fresh cycle embryo transfer was cancelled and all embryos were cryopreserved by vitrification if any of the following occurred: (1) ovarian hyper-stimulation syndrome (OHSS) was present; (2) oocyte was obtained for ≥20 to prevent OHSS; (3) embryo developed slowly in *ex vivo* culture, quality of embryos was poor, or embryos failed to develop; (4) serum P level was >2 ng/mL on day of HCG injection; (5) endometrial lesions were observed on ultrasound.

### 2.4. Statistical Analysis

Statistical analysis was conducted using SPSS26.0 (SPSS Inc., Chicago, IL, USA). Continuous variables with normal distribution are presented as mean and standard deviation and analysed using a one-way analysis of variance (ANOVA). Continuous variables not following normal distribution are presented as median and interquartile range and analysed using the Kruskal-Wallis *H* test. Bonferroni correction was conducted for pairwise comparison for both ANOVA and Kruskal-Wallis H test, with *P* < 0.008 considered statistically significant. Categorical variables are expressed as number and percentage and analysed using the chi-square test or Fisher exact test (when the expected numbers are <5), with *P* < 0.05 considered statistically significant. Forward logistic regression analysis was performed to determine the risk factors for miscarriage.

## 3. Results and Discussion

### 3.1. Results

#### 3.1.1. Demographic and Baseline Characteristics

**Figure 1:**
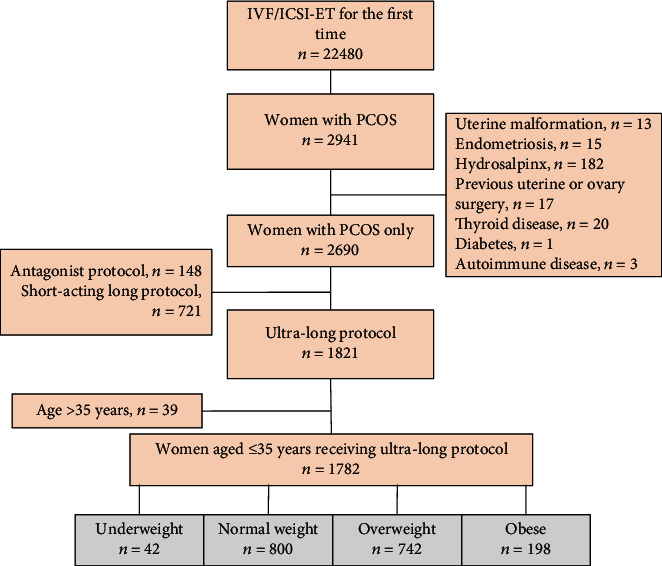
Study flow chart.

The study flowchart is shown in Figure 1. The demographic and baseline characteristics of the study women are summarized in Table 1. A total of 22,480 women underwent IVF/ICSI-ET during the study period, among which 2941 had PCOS. After excluding subjects with uterine malformation, endometriosis, hydrosalpinx, previous uterine/ovary surgery, thyroid disease, diabetes, or autoimmune disease, 2690 women with PCOS underwent IVF/ICSI-ET for the first time. We excluded 721 women who received the short-acting forms in the long protocol, and 148 women who received antagonist protocol. In addition, 39 women aged above 35 years were excluded. The final analysis included 1782 women. The mean age was 28.1 ± 3.1 years (range 21 to 35 years). The mean BMI was 25.54 ± 4.21 kg/m^2^ (range 16.53 to 42.78 kg/m^2^). 42 (2.36%) women were underweight, 800 (44.89%) women were normal weight, 742 (41.64%) women were overweight, and 198 (11.11%) were obese. The underweight group had significantly higher basal LH than the overweight and obese groups (One-way ANOVA, Bonferroni correction; *P* < 0.008). The four groups were comparable in demographic and other baseline variables including duration of infertility, basal FSH, and basal testosterone.

#### 3.1.2. Hormonal Changes and Ovary Responses during Controlled Ovarian Stimulation

There was no statistical difference in the fertilization rate with either IVF or ICSI among the four groups (Table 2). The total dose and duration of gonadotropin were significantly lower/shorter in the underweight and normal weight groups than in the overweight and obese groups (Kruskal-Wallis *H* test, Bonferroni correction; *P* < 0.008). The number of oocytes retrieved was significantly lower in the overweight and obese groups than in the normal weight group (One-way ANOVA, Bonferroni correction; *P* < 0.008). The number of oocytes retrieved was significantly lower in the overweight group than the obese group (One-way ANOVA, Bonferroni correction; *P* < 0.008). E_2_ on the day of HCG injection was significantly higher in the underweight and normal weight groups than in the overweight and obese groups (Kruskal-Wallis *H* test, Bonferroni correction; *P* < 0.008), whereas progesterone on the day of HCG injection was significantly higher in the underweight and normal weight groups than in the overweight and obese groups (One-way ANOVA, Bonferroni correction; *P* < 0.008). No significant difference was observed in LH and endometrial thickness on the day of HCG injection among the four groups. The number of transferable embryos was significantly higher in the normal weight group than in the overweight and obese groups (One-way ANOVA, Bonferroni correction; *P* < 0.008).

#### 3.1.3. Obstetric Outcomes

No statistical difference was found among the 4 groups in embryo implantation rate, clinical pregnancy rate, live birth rate, twin rate, singleton rate, preterm birth rate, full-term rate, and ectopic birth rate (Table 3). The cycle cancellation rate was significantly lower in the obese group than in the normal weight and underweight groups (Chi-square test, Bonferroni correction; *P* < 0.008). In comparison to the normal weight group, the cycle cancellation rate was significantly lower in the overweight group (Chi-square test, Bonferroni correction; *P* < 0.008). The miscarriage rate was significantly higher in the obese group than the normal weight group (Fisher's exact test, Bonferroni correction; *P* < 0.008).

Potential factors entered as independent variables in the multivariate logistic regression analysis for miscarriage included age, infertile duration, BMI, basal FSH, basal testosterone, total gonadotropin dosage, gonadotropin stimulation days, number of oocytes retrieved, E_2_ on HCG day, progesterone on HCG day, endometrial thickness and available embryos, and LH levels (both basal LH and LH on the day of HCG administration). BMI was independently associated with miscarriage (OR: 1.069, 95% CI 1.020, 1.122, *P* = 0.006) (Table 4).

## 4. Discussion

PCOS is the most common endocrine disease in women of reproductive age. Major clinical features include hypertestosteronemia, high LH, and insulinemia and menstrual irregularities, obesity, and infertility. Follicles in PCOS women undergo premature luteinization and are prone to atresia due to prolonged hormonal aberration in the internal milieu. High LH levels and insulin resistance in PCOS women affected oocyte quality, predisposing these women to increased risk of abortion and reduced live birth rate [17]. Obesity reduces the natural conception rate; however, the effect of obesity on the therapeutic outcome of IVF/ICSI remains inconclusive. Currently, many studies are available on the effects of obesity on the therapeutic outcome of IVF/ICSI in PCOS women, but they often have a limited sample size. In addition, no consensus ovarian stimulation protocol was used in these studies; therefore, they cannot accurately elucidate the effects of BMI on assisted reproductive outcome in PCOS women. Our study retrospectively analyzed the baseline data on 1782 cycles and obstetric outcome of PCOS women (aged ≤35 years) who received IVF/ICSI-ET. We found no significant difference in the clinical pregnancy rate and live birth rate of PCOS women, indicating no effect of BMI on the clinical pregnancy rate and live birth rate. However, we observed a modest but significant increase in the risk of miscarriage among PCOS women with a higher BMI versus those with a lower BMI.

The study showed that at baseline, women with lower BMI had higher LH, and underweight women had higher LH than women who were overweight or obese. LH and FSH are hormones essential for follicle development, maturation, and ovulation. The causes and mechanisms for low LH in obese women are still not clear, which could be related to higher insulin levels in women and abnormal pulsatile secretion of GnRH by the hypothalamus and LH by the pituitary gland. The total dose and duration of gonadotropin were significantly lower or shorter in the underweight group and the normal weight group than the overweight group and the obesity group, indicating that the dose and duration of gonadotropin increased with BMI. Obese women require higher doses of gonadotropin [13] for a longer duration to stimulate ovulation. Increased BMI could reduce effective serum concentration of gonadotropin and increased fatty tissues may cause increased leptin secretion [18]. These pharmacokinetic changes lead to relative gonadotropin resistance; as a result, obese PCOS women not only use gonadotropin for longer duration but also at higher doses [19]. Pretreatment before controlled ovarian stimulation is very important in overweight and obese PCOS women. After weight reduction, gonadotropin dose can be reduced and ovarian stimulation duration can be shortened, which not only facilitates oocyte retrieval but also is economical. Furthermore, the number of oocytes retrieved in the underweight group and the normal weight group was higher than that of the overweight group and the obesity group, which is consistent with most studies [18, 20], suggesting that in PCOS women, increase in BMI could negatively impact on ovulation. In addition, Metwally et al. [21] found that obesity had a negative impact on embryo quality in young women who received IVF-assisted reproduction. Studies also revealed changes in both the components of the follicular fluid and the metabolic milieu in obese women [22–25]. Moreover, obese women display spindle abnormalities and spindle disarrays in oocytes [26], which may lead to a decline in oocyte quality. In the current study, the number of transferable embryos declined as BMI increased and was significantly higher in normal weight women than overweight and obese women.

It remains inconclusive whether BMI affects the obstetric outcome of PCOS women receiving IVF/ICSI. Bellver et al. [27] reviewed data on donor ovulation cycles with all donor oocytes. In recipient women with increased BMI, the clinical pregnancy rate, embryo implantation rate, and live birth rate significantly declined. Provost et al. [28] retrospectively analyzed the clinical data of 1782 PCOS women receiving IVF/ICSI and found that embryo implantation rate and clinical pregnancy rate in women whose BMI was >30 kg/m^2^ were apparently lower than women with normal BMI. Meanwhile, compared with women with normal BMI, women whose BMI was >50 kg/m^2^ had a markedly higher early spontaneous miscarriage rate. In the current study, we also found that higher BMI was associated with a modest but significant increase in the risk of miscarriage (OR: 1.069, 95% CI 1.020, 1.122; *P* = 0.006). Normal weight PCOS women had the lowest miscarriage rate versus underweight, overweight, and obese PCOS women. The lowest miscarriage rate was approximately 30% in both underweight and obese PCOS women, suggesting that PCOS women are predisposed to abortion when BMI is too high or too low. Huang et al. [29] analyzed the IVF cycle outcome of 128 PCOS women and found that the clinical pregnancy rate of normal weight or underweight PCOS women (BMI < 24 kg/m^2^) was markedly higher than that of overweight or obese PCOS women (BMI ≥ 24 kg/m^2^). Meanwhile, Plachot et al. [17] retrospectively analyzed 1074 PCOS women receiving IVF/ICSI and found that high BMI had no effect on the obstetric outcome in Chinese PCOS women. The results of the current study revealed no statistical difference in embryo implantation rate, clinical pregnancy rate, live birth rate, twin rate, singleton rate, preterm birth rate, full-term rate, and ectopic birth rate among the four groups. The miscarriage rate was significantly higher in the obese group than the normal weight group. In multivariate logistic regression analysis, abnormal BMI was an independent risk for miscarriage.

The current study is retrospective in design. As a result, the cause-effect relationship between BMI and obstetric outcomes cannot be drawn. Also, the sample size is not sufficient to allow us to recapitulate the previously reported differences. The cumulative live birth rate was not collected in the current study since a significant proportion (approximately 20%) of the patients who failed the initial cycle with fresh embryos were lost to further follow-up. As such, the outcome measures reflect only part of the entire clinical spectrum.

In conclusion, overweight and obesity are associated with poor obstetric outcomes in PCOS patients at ≤35 years of age receiving ultra-long protocol. Weight reduction should be encouraged in such patients prior to ART.

## Figures and Tables

**Table 1:** Demographic and baseline characteristics of the study population.

	Underweight (*n* = 42)	Normal weight (*n* = 800)	Overweight (*n* = 742)	Obese (*n* = 198)	*P*
Age, years	27.76 ± 2.59	28.13 ± 3.15	28.19 ± 3.14	27.97 ± 3.03	0.713
Infertile duration (y), median (range)	3 (2, 5)	3 (2, 4)	3 (2, 5)	4 (2, 5)	0.061
Basal FSH (mIU/mL)	6.89 ± 1.19	6.36 ± 1.24	6.12 ± 1.25	6.08 ± 1.28	0.819
Basal LH (mIU/mL)	10.12 ± 5.05	8.69 ± 4.44	8.15 ± 4.36^∗^	7.90 ± 4.33^∗^	<0.001
Basal testosterone (ng/mL)	0.76 ± 0.36	0.70 ± 0.34	0.68 ± 0.35	0.66 ± 0.32	0.232

Data are presented as mean and standard deviation unless stated otherwise. FSH: follicle-stimulating hormone; LH: luteinizing hormone. ^∗^ significantly different vs. the underweight group.

**Table 2:** Characteristics of ovarian responses and laboratory data.

	Underweight (*n* = 42)	Normal (*n* = 800)	Overweight (*n* = 742)	Obese (*n* = 198)	*P*
Fertilization rate (%)					
IVF	29 (69%)	608 (76%)	552 (74.4%)	152 (76.8%)	0.649
ICSI	13 (31%)	192 (24%)	190 (25.6%)	46 (23.2%)	0.649
Total Gn dosage (IU)	1950 (1474, 2459)	2175 (1650, 2625)	2637 (2100, 3375)^∗^	3375 (2629, 4247)^∗∗^	0.001
Gn stimulation, days	12 (11, 13)	12 (10, 13)	12 (11, 15)^∗^	14 (12, 17)^∗∗^	0.001
Oocytes retrieved (*n*)	18.20 ± 6.15	19.16 ± 8.55	16.71 ± 8.27^∗∗∗^	14.95 ± 7.83^∗∗^	0.001
E_2_ on HCG day (pg/mL)	4936 (4830, 5060)	4839 (3688, 4947)^∗∗∗∗^	4207 (2628, 4936)^∗∗^	3576 (2303, 4936)^∗∗^	0.001
Progesterone on HCG day (ng/mL)	1.39 ± 0.59	1.23 ± 0.55	1.11 ± 0.50^∗^	1.07 ± 0.52^∗^	0.001
LH on HCG day (mIU/mL)	1.03 (0.61, 1.46)	0.99 (0.71, 1.32)	0.93 (0.68, 1.22)	0.92 (0.69, 1.19)	0.074
Endometrial thickness	10.59 ± 1.87	10.88 ± 1.94	10.96 ± 1.93	11.19 ± 1.88	0.151
Available embryos	5.81 ± 3.44	5.75 ± 3.91	5.01 ± 3.56^∗∗∗^	4.84 ± 3.24^∗∗∗^	0.001

Continuous variables are presented as either mean ± standard deviation or median (interquartile range). Categorical variables are expressed as number and percentage. IVF: in vitro fertilization; ICSI: intracytoplasmic sperm injection; Gn: gonadotropin; E_2_: estradiol; LH: luteinizing hormone; HCG: human chorionic gonadotropin. ^∗^ significantly different vs. the underweight group and normal weight group. ^∗∗^ significantly different vs. the underweight group and normal weight group. ^∗∗∗^ significantly different vs. the normal weight group. ^∗∗∗∗^ significantly different vs. the normal weight group. ^∗^, ^∗∗^, ^∗∗∗^, and ^∗∗∗∗^ were significantly different.

**Table 3:** Pregnancy outcomes.

	Underweight (*n* = 42)	Normal weight (*n* = 800)	Overweight (*n* = 742)	Obese (*n* = 198)	*P*
Embryo transfer cancellation	66.7% (28/42)	57.8%^∗^ (462/800)	49.2% (365/742)	38.9%^∗∗^ (77/198)	<0.001
Embryo implantation	39.3% (11/28)	47.0% (319/679)	47.5% (358/753)	48.3% (115/238)	0.834
Clinical pregnancy	71.4% (10/14)	63.6% (215/338)	66.6% (251/377)	66.9% (81/121)	0.810
Miscarriage	30.0% (3/10)	12.6% (27/215)	19.5% (49/251)	29.6%^∗∗∗^ (24/81)	0.004
Live-birth	42.9% (6/14)	53.0% (179/338)	50.1% (189/377)	46.3% (56/121)	0.567
Singleton pregnancy	80.0% (8/10)	53.5% (115/215)	56.6% (142/251)	55.6% (45/81)	0.435
Twin pregnancy	10.0% (1/10)	46.1% (99/215)	42.2% (106/251)	43.2% (35/81)	0.149
Preterm delivery	0.0% (0/10)	21.8% (39/179)	23.3% (44/189)	35.7% (20/56)	0.103
Full-term delivery	100% (6/6)	78.2% (140/179)	76.7% (145/189)	64.3% (36/56)	0.103
Ectopic pregnancy	7.1% (1/14)	0.3% (1/338)	0.8% (3/377)	0.8% (1/121)	0.096

Data are expressed as percentage (number). ^∗^ significantly different vs. the overweight group and obese group. ^∗∗^ significantly different vs. the underweight and normal weight groups. ^∗∗∗^ significantly different vs. normal weight groups. ^∗^, ^∗∗^, and ^∗∗∗^ were significantly different.

**Table 4:** Multivariate logistic regression analysis of risk factors for miscarriage.

	OR	95% CI	*P*
Age, years	0.967	0.895-1.045	0.396
Infertile duration, years	1.048	0.948-1.158	0.361
BMI, kg/m^2^	1.069	1.020-1.122	0.006
Basal FSH (mIU/mL)	0.896	0.745-1.078	0.245
Basal LH (mIU/mL)	0.977	0.927-1.031	0.397
Basal testosterone (ng/mL)	0.652	0.324-1.311	0.230
Total Gn dosage (IU)	1.000	1.000-1.000	0.555
Gn stimulation, days	1.067	0.952-1.195	0.267
Oocytes retrieved (n)	0.969	0.917-1.024	0.269
E_2_ on HCG day (pg/mL)	1.000	1.000-1.000	0.959
Progesterone on HCG day (ng/mL)	0.903	0.560-1.455	0.674
LH on HCG day (mIU/mL)	0.776	0.458-1.317	0.348
Endometrial thickness	1.000	0.889-1.126	0.996
Available embryos	0.981	0.892-1.078	0.685

BMI: body mass index; FSH: follicle-stimulating hormone; LH: luteinizing hormone; Gn: gonadotropin; E_2_: estradiol; HCG: human chorionic gonadotropin. CI: confidence interval; OR: odds ratio.

## Data Availability

The data used to support the findings of this study are available from the corresponding author upon request.
